# Calcitonin gene-related peptide is a potential autoantigen for CD4 T cells in type 1 diabetes

**DOI:** 10.3389/fimmu.2022.951281

**Published:** 2022-09-16

**Authors:** Wei Li, Ronghui Li, Yang Wang, Yan Zhang, Munendra S. Tomar, Shaodong Dai

**Affiliations:** ^1^ Department of Pharmaceutical Sciences, Skaggs School of Pharmacy and Pharmaceutical Sciences, University of Colorado Anschutz Medical Campus, Aurora, CO, United States; ^2^ National Health Commission (NHC) Key Laboratory of Pulmonary Immune-related Diseases, Guizhou Provincial People’s Hospital, Guiyang, China; ^3^ Department of Immunology and Microbiology, School of Medicine, University of Colorado Anschutz Medical Campus, Aurora, CO, United States

**Keywords:** type 1 diabetes (T1D), CGRP, MHC, CD4 T cells, thiol regulation

## Abstract

The calcitonin gene-related peptide (CGRP) is a 37-amino acid neuropeptide with critical roles in the development of peripheral sensitization and pain. One of the CGRP family peptides, islet amyloid polypeptide (IAPP), is an important autoantigen in type 1 diabetes. Due to the high structural and chemical similarity between CGRP and IAPP, we expected that the CGRP peptide could be recognized by IAPP-specific CD4 T cells. However, there was no cross-reactivity between the CGRP peptide and the diabetogenic IAPP-reactive T cells. A set of CGRP-specific CD4 T cells was isolated from non-obese diabetic (NOD) mice. The T-cell receptor (TCR) variable regions of both α and β chains were highly skewed towards TRAV13 and TRBV13, respectively. The clonal expansion of T cells suggested that the presence of activated T cells responded to CGRP stimulation. None of the CGRP-specific CD4 T cells were able to be activated by the IAPP peptide. This established that CGRP-reactive CD4 T cells are a unique type of autoantigen-specific T cells in NOD mice. Using IA^g7^-CGRP tetramers, we found that CGRP-specific T cells were present in the pancreas of both prediabetic and diabetic NOD mice. The percentages of CGRP-reactive T cells in the pancreas of NOD mice were correlated to the diabetic progression. We showed that the human CGRP peptide presented by IA^g7^ elicited strong CGRP-specific T-cell responses. These findings suggested that CGRP is a potential autoantigen for CD4 T cells in NOD mice and probably in humans. The CGRP-specific CD4 T cells could be a unique marker for type 1 diabetes. Given the ubiquity of CGRP in nervous systems, it could potentially play an important role in diabetic neuropathy.

## Introduction

**Figure 1:**
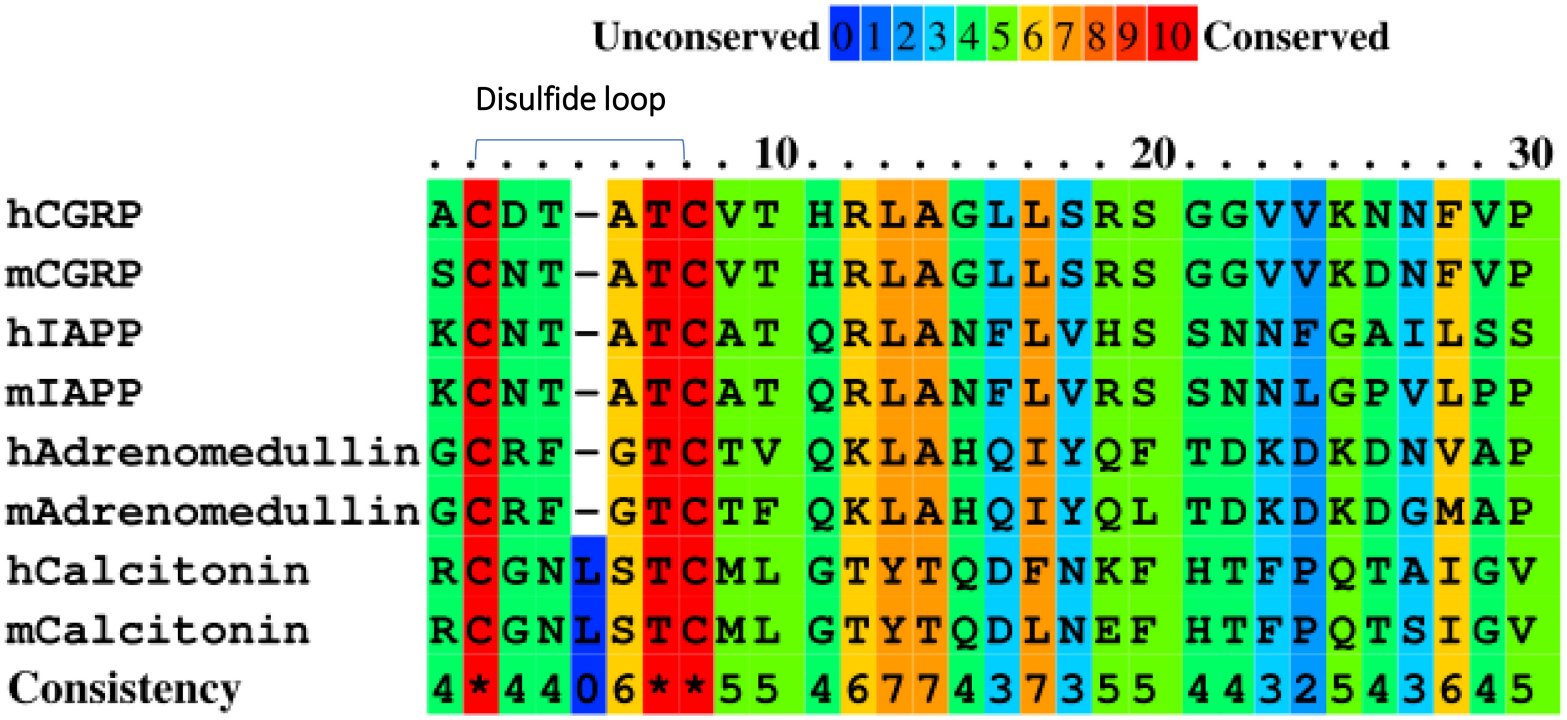
Amino acid sequence alignment of the CGRP family proteins in humans (h) and mice (m) using PRALINE.

In type 1 diabetes (T1D), the autoantigens in the pancreas are presented by major histocompatibility complex (MHC) molecules that activate diabetogenic T cells and play important roles in the destruction of pancreatic insulin (Ins)-producing β cells. Many autoantigens have been characterized as targets of T cells, including proinsulin, a 65-kDa isoform of glutamate decarboxylase (GAD65), insulinoma-associated antigen 2 (IA-2), islet-specific glucose-6-phosphatase catalytic-subunit-related protein (IGRP) (1, 2), cation efflux transporter ZNT8 (3), chromogranin A (ChgA), and IAPP. Some β-cell secretory granule proteins, such as Ins, ChgA, and IAPP, were identified as the ligands of highly pathogenic CD4 T cells in non-obese diabetic (NOD) mice (4–6) and provided essential targets for the study of T1D onset, diagnosis, and therapy. The CGRP family is a group of peptide hormones consisting of IAPP, calcitonin, adrenomedullin, and CGRP. Although sequence homology among the members of this family ranges between 20% and 50%, they all have a conserved N-terminal disulfide bridge and the amidated C-terminus (7) (**Figure 1**). IAPP is derived from an 89-residue prohormone precursor to form the mature 37-amino-acid peptide hormone. IAPP was the first member of the CGRP family proteins recognized as an essential contributor to β-cell dysfunction (8, 9). The pathogenic functions of IAPP as an autoantigen in T1D emerged promptly (6). Recently, the epitopes of the well-studied diabetogenic CD4 T-cell clones, namely, BDC-5.2.9 and BDC-6.9, were identified as IAPP N-terminal peptides (KS20) and IAPP-Ins hybrid peptides, respectively (5, 10). Like IAPP, the processed CGRP is a 37-amino-acid peptide concentrated in sensory nerve endings and widely expressed in neuronal tissue. It has a significant role in sensory neurotransmission. Inhibitory monoclonal antibodies (mAbs) targeting CGRP showed the efficiency of migraine alleviation and were recently approved by the Food and Drug Administration (FDA) for migraine treatment (11). CGRP is co-localized with Ins and IAPP in the Ins-secreting β cells in the pancreas (12, 13), which can inhibit the production of interleukin (IL)-2, tumor necrosis factor (TNF)-α, TNF-β, and interferon gamma (IFN-γ) by T lymphocytes *in vitro* (14, 15). It has been reported that CGRP could promote Th17 T-cell-mediated autoimmune inflammation by regulating IL-17 expression (16). The expression level of CGRP decreased significantly in pancreatic islet cells of diabetic patients compared to the controls (17). Armen *et al.* found that CGRP was a potential therapeutic molecule to treat diabetes and reported that increased expression of CGRP to β cells decreased the incidence of T1D in female NOD mice (18). There are conflicting reports arguing if CGRP may inhibit or stimulate Ins secretion (19–21).

The KS20 peptide is the first 20 amino acids of the processed IAPP peptide. It is delineated as the epitope of the prototypic diabetogenic T-cell line, BDC-5.2.9 (5). IAPP KS20-reactive T cells could be detected in the pancreas of prediabetic and diabetic NOD mice and contribute to disease development (22). So far, there are no reports of CGRP-reactive T cells in the pancreas of NOD mice. CGRP is present in the pancreas and nervous system and shares biological activity with IAPP. Given the similar amino acid sequence of N-terminal segments between CGRP and IAPP, we expected that the CGRP-reactive CD4 T cells would exist in the pancreas of NOD mice and contribute to the development of T1D. The disulfide bridge of the IAPP KS20 peptide play an essential role in the activation of BDC-5.2.9 (22, 23). We speculated that the N-terminal 20 amino acids of CGRP might also cross-activate BDC-5.2.9. In this study, we demonstrated that CGRP cannot cross-activate BDC-5.2.9. Using the IA^g7^-linked CGRP tetramers, we identified CGRP-reactive T cells in the pancreas of prediabetic and diabetic NOD mice. We then generated a panel of CGRP-specific T-cell hybridomas and determined TCR V gene segment usage. The results paved the way to determine the role of CGRP-specific T cells in the development of T1D and diabetes-related neuropathy.

## Materials and methods

### Mice

Female NOD/LtJ mice and BALB/c mice were purchased from the Jackson Laboratories and maintained in the Biological Resource Center at National Jewish Health (Denver, CO). Prediabetic NOD mice used in this study were 12 weeks of age and had normal blood glucose readings before experiments (<250 mg/dl). Mice were considered diabetic when blood glucose levels were >250 mg/dl for two consecutive days. Animal husbandry and experimental procedures were conducted under protocols approved by the Institutional Animal Care and Use Committee of the National Jewish Health.

### Reagents

Fluorescently labeled mAbs for flow cytometry were used as follows: FITC-B220, eFluor 450-F4/80, APC eFluor 780-CD8, Alexa Fluor 700-CD4, APC-Foxp3, and PerCP-Cy5.5-CD44 (eBioScience), and Foxp3/Transcription Factor Fixation/Permeabilization (eBioscience, 501129060). Unlabeled TCR Cβ-specific H57-597 was expressed and purified from the B-cell hybridoma (a gift from Kappler/Marrack lab). All peptides were synthesized by Peptide 2.0 Inc.

### IA^g7^-CGRP and IAPP tetramer preparation

The first 20 amino acids (SCNTATCVTHRLAGLLSRSG, SG20) of mouse CGRP peptide (equivalent to KS20 peptide) are similar to the first 20 amino acids (ACDTATCVTHRLAGLLSRSG, AG20) of human CGRP peptide (5). The IA^g7^ α and β chains were inserted in the baculovirus transfer vector under P10 and PH promoters, respectively. The β chain contained the wild-type (WT) CGRP SG20 peptide, or a CGRP variant SG20S17E (SCNTATCVTHRLAGLL **E** RSG), or the IAPP KS20V17E peptide (KCNTATCATQRLANFL **E** RSS) or the truncation of IAPP KS20 peptide from 1 to 5, KS20△1–5 peptide (TCATQRLANFL **E** RSS), with a C-terminal flexible linker (GGGSLVPRGSGGGGS) inserted between a signal peptide and the N terminus of the β1 domain as previously described (24, 25). All constructs contained this flexible linker attaching the peptide to the N terminus of the IA^g7^ β chain and a stabilizing acid–base leucine zipper attached to the C terminus of the IA^g7^ (26). The crystal structure of the IA^g7^-KS20 complex showed that the position of 17 amino acids occupied the P9 binding pockets of IA^g7^ (data not shown). Hence, we introduced a mutation at position 17 to E (underlined) to enhance the binding between the peptide and IA^g7^ without adverse effects on the peptide activity. The soluble IA^g7^-SG20, IA^g7^-KS20, and IA^g7^-KS20△1–5 complexes were produced and purified as previously described (25). Briefly, Hi5 cells were infected separately with IA^g7^-SG20, IA^g7^-KS20, or IA^g7^-KS20△1-5 recombinant baculovirus in spinner flasks at 19°C for 6 days. The supernatants were harvested, and the debris was removed by centrifugation. Soluble IA^g7^-SG20, IA^g7^-KS20, and IA^g7^-KS20△1–5 were purified using zipper-specific 2H11 mAb (27) and coupled with CNBr-activated Sepharose 4B resins (GE Healthcare). As previously described, the purified proteins were biotinylated with the BirA enzyme (28). After biotinylation, the IA^g7^-SG20 protein was applied to a Superdex 200 Increase column (GE Healthcare) to remove the aggregate or other contaminating proteins, and peak 2 was collected for the tetramer generation (**Supplementary Figure S1A**). The purified biotinylated protein was analyzed by sodium dodecyl sulfate–polyacrylamide gel electrophoresis (SDS-PAGE) with and without reduction (**Supplementary Figure S1B**). These biotinylated proteins were mixed with streptavidin-PE for 1 h at 4°C and then were purified again with a Superdex 200 Increase column (**Supplementary Figure S1C**). The IA^g7^-KS20 tetramers and IA^g7^-KS20△1–5 tetramers were produced with the same method.

### The construction of KS20 and SG20 mutants

The plasmids of IA^g7^-KS20 and IA^g7^-SG20 containing the transmembrane-cytoplasmic tail of the baculovirus gp64 protein were used as templates for mutation. The primers were used for mutation in **Supplementary Table S1**. Site-directed mutagenesis experiments were carried out using PfuUltra II fusion DNA polymerase (Agilent Technologies). The PCR reaction system is 50 μL: 5 μL of 10× reaction buffer, 50 ng of DNA template, 1 μL of 10 μM forward and reverse primer, 1 μL of dNTP, 1 μL of PfuUltra II, 3 μL of dimethyl sulfoxide (DMSO), and water added to the final volume of 50 μL. The temperature program used was 1 min at 95°C followed by 18 cycles of 50 s at 95°C, 50 s at 60°C, and 8 min at 68°C. The PCR product was digested with 1 μL DpnI (R0176L, NEB) for 2 h and transformed to competent DH5α.

### T-cell stimulation assay

For antigen-presenting cells (APCs), we used two forms of the M12.C3 B-cell lymphoma, one expressing IA^g7^ (M12C3^G7^) and the other expressing DQ8 (M12C3-DQ8) (29). T-cell hybridomas (1×10^5^ cells/well) were mixed with an equal amount of APCs and cultured overnight with various concentrations of KS20 or SG20 peptides in a volume of 250 µL. Twenty-four hours later, culture supernatants were then harvested, and secreted IL-2 was measured with a functional assay, following the growth and survival of the HT-2, which is an IL-2-dependent cell line (30). For insect cell surface expression, the different viruses encoding WT KS20, KS20 variants, WT SG20, or SG20 mutants were used to infect mouse ICAM^+^B7^+^ SF9 insect cells (28). Three days later, cells in each well were washed with balanced salt solution (BSS) two times and used as APCs to stimulate BDC-5.2.9 or CGRP-reactive T-cell hybridomas (1×10^5^ cells/well). Twenty-four hours later, supernatants were collected for HT-2 assay.

### ELISA for binding assays

Briefly, 96-well plates were first coated with 100 μg/ml streptavidin overnight at 4°C. After blocking with 30% fetal bovine serum (FBS) and washing three times with PBS-T (0.1% Tween in phosphate-buffered saline, PBS), 5 μg/mL biotinylated protein IA^g7^-KS20, IA^g7^-SG20, or IA^g7^-SG20C7S was added and incubated for 1 h at 37°C. After washing with PBS-T, 100 μL of different concentrations of mAb LD96.24 was added and incubated for 1 h at room temperature. Then, IgG-alkaline phosphatase (1:30,000) was added and incubated for another hour at room temperature. After five times washing with PBS-T, p-nitrophenyl phosphate (PNPP) substrate solution was added and incubated for 30–60 min, and then, the OD_405_ was measured.

### Preparation of pancreatic cells of NOD mice

Pancreatic cells were prepared following the protocol as described previously (31). Briefly, freshly isolated NOD pancreases were cut into small pieces and digested in 25 mL of BSS containing 5% (vol/vol) FBS plus 5 μM CaCl_2_ and 100 μg/mL collagenase (C9407; Sigma) at 37°C for 15 min. The digested mixture was then washed with 10% FBS in BSS, crushed, and passed through a 100-μm nylon mesh screen. The cells were washed multiple times in 10% FBS in BSS until the supernatant was clear and then resuspended in 10% FBS in spinner-minimum essential medium (S-MEM) (Gibco).

### Flow cytometry analysis of tetramer-positive T cells in the pancreas

For tetramer staining, we followed the protocol as previously described (28). Briefly, approximately 20 μg/mL MHC II-Peptide tetramers and 1 μg/mL H57-597 were added at 25 μL/well of 96-well U plates and then incubated at 37°C in a 5% CO_2_ incubator for 2 h. After tetramer staining and cell surface staining with fluorescent anti-B220, anti-F4/80, anti-CD8, anti-CD44, and anti-CD4 mAbs, cells were fixed and intracellular staining with Foxp3-APC using the eBioscience Foxp3/Transcription Factor Staining Buffer Set (Thermo Fisher Scientific) according to the manufacturer’s instructions. Stained single-cell suspensions were analyzed using a Fortessa flow cytometer running FACSDiva (BD Biosciences, US). FSC 3.0 files were analyzed with Flowjo 10.0 Software.

### Generation of CGRP-specific T-cell hybridomas

CGRP-specific T-cell hybridomas used in these studies were generated as described previously (30). Briefly, three female NOD mice were immunized with 10 μg lipopolysaccharide (LPS) and 100 μg CGRP SG20 peptide by intraperitoneal injection (i.p) twice at 2-week intervals. Four days after the final challenge, pancreatic lymph nodes (PLNs) and splenocytes were isolated and re-stimulated with 10 μg/mL CGRP SG20 peptide for 4 days. Cells were purified with lymphocyte separation medium (MP Biomedicals) and cultured in a complete tumor medium (CTM) containing IL-2 for 3 days. T-Cell hybridomas were established by fusion of the purified T-cell blasts to the thymoma BW5147α^−^β^−^ thymoma cells using polyethylene glycol 1450 and cloned by limiting dilution under HAT (Sigma, H0262) selection as described previously (30). For CGRP-specific T-cell screening, T-cell hybridomas were stimulated by M12C3^G7^ with or without 1 μg/mL SG20S17E, and supernatants of each well were collected for IL-2 assay 24 h later. The positive wells were CGRP-specific T-cell hybridomas, and they were used for the following experiments.

### Determination of TCR variable regions of both α and β chains

Total RNA of each CGRP-reactive T-cell hybridomas was isolated, and cDNA was prepared using PrimeScript™ RT Reagent Kit (RR037A, Takara). We first used the FITC-anti-TCR Vβ 1-14 (BioLegend) to stain CGRP-specific T-cell hybridomas to determine TCR Vβ segment usages. Then, TCR Vβ gene fragments were PCR amplified by applying a TCR C region primer of the β chain and a TRBV primer according to the flow results. We used a TCR C region primer of the α chain and a panel of TRAV primers (n=13) to determine TRAV gene segment usage. Upon determination of specific TRAV and TRBV gene segment usages, larger-scale PCR reactions were purified with the Gel and PCR Clean-UP kit (Takara Bio USA, Cat. No. 740609.250). The PCR products were ligated to pCR™2.1 Vector using TA Cloning™ Kit (Invitrogen, Cat. No. K202020). After transforming DH5α cells, the single clone was selected for sequencing. All TRAV and TRBV sequences were analyzed using ImMunoGeneTics (IMGT)/HighV-QUEST (http://www.imgt.org).

### Statistical analyses

Comparisons between two groups were performed with a two-tailed Student’s t-test, and more than two groups were compared by ANOVA. Each experiment was repeated at least once to assess reproducibility. *p < 0.05, **p < 0.01, ***p < 0.001.

## Results

### No cross-reactivity between CGRP and IAPP-specific T cell BDC-5.2.9

**Figure 2:**
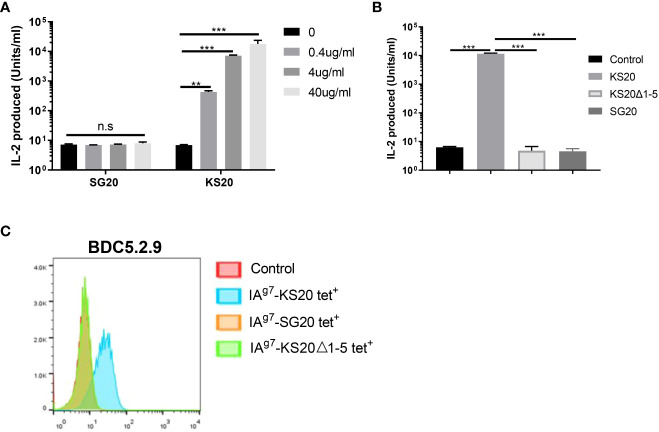
The cross-reaction between the CGRP peptide and IAPP reactive T cell, BDC5.2.9. **(A)** M12C3G7 incubated with SG20 or KS20 peptides stimulated BDC5.2.9 at different concentrations, 0, 0.4, 4 and 40μg/mL. 24 hours later, supernatants were collected for HT-2 assay. Results are the means ± SEM of triplicate wells. **(B)** ICAM+B7^+^ SF9 insect cells expressing IA^g7^-WT KS20, IA^g7^-KS20△1-5, or IA^g7^-SG20 were used as APCs to stimulate BDC5.2.9 T cells. Results are the means ± SEM of triplicate wells. **(C)** IA^g7^-SG20 tetramers were used to stain BDC5.2.9. IA^g7^-KS20 was used as a positive control and IA^g7^-KS20△1-5, as a negative control. *P< 0.05, **P< 0.01, ***P< 0.001.

CGRP family peptides all share a common motif CXXXXC near the N-terminus both in human and mice (**Figure 1**) (32). The mouse CGRP and mouse IAPP contain the same CNTATC motif. There is only one amino acid difference between human CGRP and mouse CGRP CXXXXC motifs. The processed CGRP peptides are present in the Ins-secreting β cells and are similar to IAPP, with about 50% amino acid sequence homology. Both peptides are 37 amino acids long and have an N-terminal disulfide bridge between residues Cys2-Cys7. Although IAPP and CGRP peptides have been shown to have distinct functions, they have similar effects on peripheral Ins resistance. Diabetogenic T cells recognize the antigenic peptides derived from β-cell proteins presented by MHC proteins on APCs (33). IAPP and CGRP peptides are processed similarly, and CGRP may cross-activate IAPP-reactive CD4 T cells in the pancreas of NOD mice. The disulfide-containing IAPP KS20 peptide is a target antigen for highly diabetogenic CD4 T cell, BDC-5.2.9 (5, 22). The typical role of IAPP and CGRP in the immunomodulation of CD4 T-cell responses might be linked to the sequence identity of the N-terminal portion of peptides. To confirm our hypothesis, we used the B-cell line M12C3^G7^, which expresses IA^g7^ to stimulate BDC-5.2.9 in the presence of CGRP or IAPP peptides (29). ICAM^+^B7^+^ SF9 cells expressing IA^g7^-linked CGRP or IAPP peptides were also used as APCs to test their ability to stimulate BDC-5.2.9 (29). The results showed that neither CGRP SG20 peptide nor truncated IAPP KS20△1-5 peptide stimulated T-cell BDC-5.2.9 (**Figures 2A, B**). Similarly, we expressed the soluble IA^g7^-CGRP proteins and generated IA^g7^-SG20 tetramers. The IA^g7^-KS20△1-5 tetramers completely lost the binding ability to BDC-5.2.9. Like KS20△1-5, IA^g7^-SG20 tetramers did not stain BDC-5.2.9 (**Figure 2C**). These results confirmed that the KS20 disulfide bridge plays an important role in the activation of T cell BDC-5.2.9, but there is no cross-reactivity between IA^g7^-SG20 and T cell BDC-5.2.9.

### The key amino acids of KS20 peptide for the activation of BDC-5.2.9 compared with SG20 peptide

**Figure 3:**
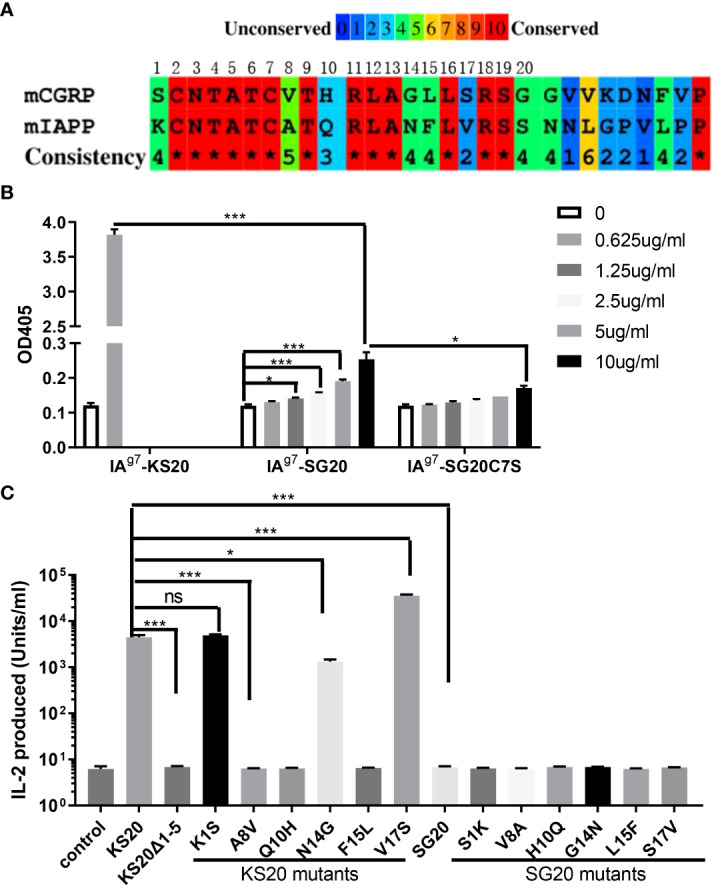
The essential amino acids of KS20 peptide for the activation of T cell BDC5.2.9 compared with the SG20 peptide. **(A)** Amino acid sequence alignment of mouse CGRP and IAPP segments studied here. **(B)** mAb LD96.24 bound weakly to IA^g7^-SG20 molecules. The binding between mAb LD96.24 and IAg7-SG20 molecules was measured by ELISA. Results are the means ± SEM of triplicate wells. **(C)** Viruses encoding WT KS20, WT SG20, KS20 mutants, and SG20 mutants were used to infect mouse ICAM^+^B7^+^ SF9 insect cells. KS20△1-5 and non-infected wells were used as controls. 3 days later, these insect cells were used as APCs to stimulate BDC5.2.9 T cells. Results are the means ± SEM of triplicate wells. *P < 0.05, **P < 0.01, ***P < 0.001.

There are seven amino acid variations between mouse IAPP KS20 and CGRP SG20 peptides. Although the disulfide loop of CGRP at the N terminal is very similar to IAPP (**Figure 3A**), the CGRP peptide did not activate the KS20-reactive T cell BDC-5.2.9. Recently, we generated a mAb, LD96.24, which binds IA^g7^-KS20 complex but not other IA^g7^-peptide complexes (23). We tested if mAb LD96.24 could bind to the IA^g7^-SG20 complex. The ELISA results showed that LD96.24 has a weaker binding affinity to IA^g7^-SG20 than IA^g7^-KS20. After mutating the Cys7 of SG20 to Ser to break the disulfide bridge, there was no binding between LD96.24 and IA^g7^-CGRP, which suggested that LD96.24 interacted primarily with the CGRP disulfide loop (**Figure 3B**). However, the same disulfide bridges present in SG20 N terminus were insufficient for BDC-5.2.9 activation. This outcome indicated that the amino acids other than the CXXXXC motif also seemed to contribute to BDC-5.2.9 T-cell stimulation. want to test which amino acids of the CGRP SG20 peptide are critical for BDC-5.2.9 activation. The differences between IAPP KS20 and CGRP SG20 peptides were at amino acid positions 1, 8, 10, 14, 15, and 17 (**Figure 3A**). Variations of these amino acids can reveal the key amino acids of IAPP and CGRP peptides affecting BDC-5.2.9 activation. We used ICAM^+^B7^+^ SF9 cells expressing IA^g7^ linked with WT KS20, and KS20 mutants of K1S, KS20 A8V, Q10H, N14G, F15L, and V17S, based on SG20 sequence as APCs to stimulate BDC5.2.9 T cells (22). The results showed that A8, Q10, and F15 were necessary for BDC-5.2.9 activation aside from the disulfide bond. The amino acid at these positions may be involved in TCR binding. Meanwhile, K1, N14, and V17 had no effects on T-cell activation. These amino acids could either be anchor residues, whose side chains were buried in the peptide-binding pockets of the IA^g7^ (34) or not in the TCR binding footprint. We also mutated CGRP SG20 to the KS20 amino acids at the same positions, including SG20 V8A, H10Q, and L15F, to see which amino acids of SG20 mutation could restore the stimulation of BDC-5.2.9. The results confirmed that amino acids of A8, Q10, and F15 were essential for T-cell BDC-5.2.9 stimulation (**Figure 3C**). However, none of these single mutations alone were able to stimulate BDC-5.2.9. The SG20 S1K, G14N, and S17V mutations were also not sufficient to restore the stimulation of SG20 to BDC-5.2.9. The data explained why SG20 did not activate T cell BDC-5.2.9.

### Detection of CGRP-reactive T cells in the pancreas of prediabetic and diabetic NOD mice

**Figure 4:**
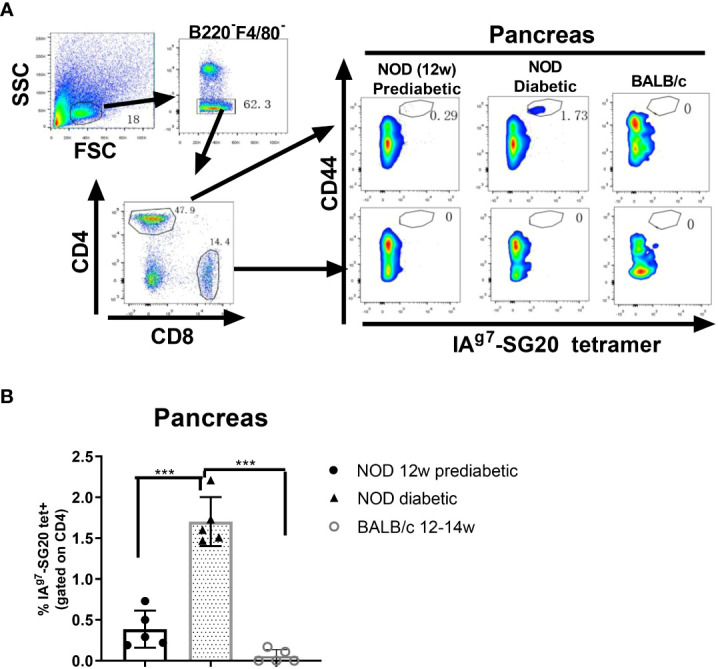
IA^g7^-SG20 tet^+^ cells were present in the pancreas of prediabetic and diabetic NOD mice. Single-cell suspensions were prepared from the whole pancreas of each prediabetic (n=5; 12 weeks) or diabetic NOD mice (n=5; 12-23 weeks). Female BALB/c mice (n=5; 12-14 weeks) were used as the negative control. The cells were stained with IA^g7^-SG20 tetramers, anti-B220, anti-F4/80, anti-CD44, anti-CD4, and anti-CD8. For tetramer analysis, cells were pregated on live, B220−, F4/80−, CD8−, CD4^+^, and CD44^high^ cells. **(A)** Representative scatter diagram of IA^g7^-SG20 tet^+^CD44^+^ cells in different groups. **(B)** The percentages of IAg7-SG20 tet+CD44+ in CD4+ T cells of each group. Each symbol represented an individual mouse. Results are the means ± SEM. *P < 0.05, **P < 0.01. ***P < 0.001.

The IA^g7^-SG20 proteins were biotinylated and incorporated into phycoerythrin (PE) streptavidin fluorescent tetramers. We used IA^g7^-SG20 tetramers to investigate whether CGRP-reactive CD4 T cells are present in the pancreas of prediabetic and diabetic NOD mice. IA^g7^-KS20 tetramers and IA^g7^-Ins B:9-23 P8G tetramers were used as positive controls (28) because these Ins and KS20-reactive pathogenic T cells were known to be present in the pancreases of prediabetic NOD mice (22, 26, 31). We examined the IA^g7^-SG20 tetramer^+^CD4^+^CD44^high^ T cells in the pancreases and PLNs of 12-week-old prediabetic NOD mice and diabetic NOD mice. Pancreatic and PLN lymphocytes were analyzed by flow cytometry, first gating on live, B220^−^, F4/80^−^, CD8^−^, CD44^high^, and CD4^+^ T cells; then, the tetramer positive cells were examined as previously described (31). The results showed that the high avidity SG20 tetramer-positive cells were easily detected in the CD44^high^ CD4^+^, but not CD8^+^ T cells with the IA^g7^-SG20 tetramer from pancreases of prediabetic and diabetic NOD female mice (**Figure 4A**). The percentages of CGRP-reactive T cells in the pancreas of diabetic mice were significantly higher than those in prediabetic NOD mice (**Figure 4B**). There are fewer CGRP-reactive CD4 T cells in PLNs than in the pancreas of prediabetic and diabetic NOD mice. The percentages of CGRP-reactive T cells were significantly higher in the PLNs of diabetic than in prediabetic NOD mice (**Supplementary Figures S2A, B**). Using the same gating strategy, KS20 and Ins B:9-23 P8G-reactive T cells in the pancreas of diabetic NOD mice were recognized as positive controls (**Supplementary Figure S3**). CGRP-reactive CD4 T cells accumulated in the pancreas of prediabetic and diabetic NOD mice, which indicated that CGRP is a potential autoantigen in NOD mice. In the meantime, we did not find any CGRP-reactive T cells in BALB/c mice. We found that CGRP-specific regulatory T cells (Tregs) exist in the pancreas of some prediabetic and diabetic NOD mice (**Supplementary Figure S4**). Tregs play an important role in suppressing T1D by controlling autoreactive T cells (35). The CGRP-reactive T cells can be divided into Foxp3^+^ and Foxp3^−^ T cells, which may play different functions in the development of T1D.

### CGRP-specific T-cell hybridomas did not cross-react with KS20 peptide

**Figure 5:**
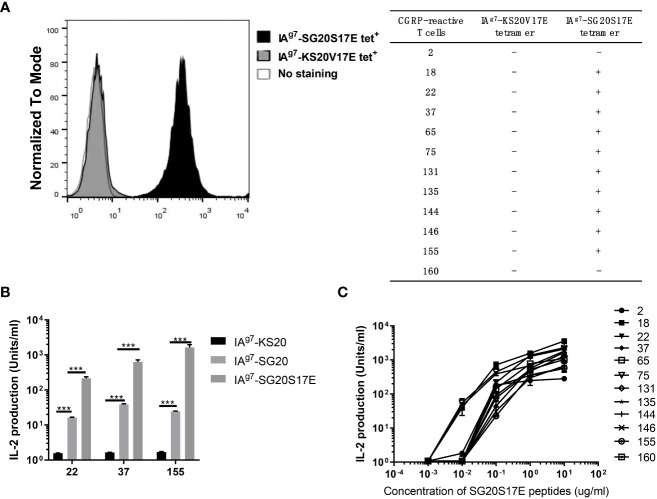
CGRP-specific T cell hybridomas did not cross-react with the KS20 peptide. 20 CGRP-specific T cell hybridomas were generated as described in materials and methods. **(A)** IA ^g7^ -SG20S17E tetramers and IA ^g7^ -KS20V17E tetramers were used to stain these CGRP reactive T cell hybridomas and unstained T cells as the negative control. The table showed the tetramer binding with the CGRP reactive T cell hybridomas. “-” represents no binding; “+” represents binding significantly. **(B)** ICAM ^+^ B7 ^+^ SF9 insect cells expressing IA ^g7^ -WT SG20 or IA ^g7^ -SG20S17E were used as APCs to stimulate three representative CGRP-reactive T cell hybridomas 22, 37, and 155. Results are the means ± SEM of triplicate wells. **(C)** M12C3 ^G7^ cells incubated with different concentrations of SG20S17E peptide stimulated the CGRP reactive T cell hybridomas. *P < 0.05, **P < 0.01, ***P < 0.001.

To further characterize CGRP-reactive T cells, we generated 20 CGRP-reactive T-cell hybridomas (20% of total hybridomas) from CGRP peptide immunized NOD mice. Most of these T-cell hybridomas could be stained by IA^g7^-SG20S17E tetramers but not IA^g7^-KS20V17E tetramers (**Figure 5A**), which indicated that these SG20-specific T cells could not cross-react with the KS20 peptide. Due to the similarity of SG20 and KS20 N termini, we evaluated if the SG20-specific T-cell clones 22, 37, and 155 respond to IAPP KS20 peptide with different TCR V segment usages. ICAM^+^B7^+^ SF9 expressing WT SG20, KS20, or SG20S17E peptides were used for T-cell stimulation assay. The results affirmed that the 22, 37, and 155 CGRP T cell hybridomas did not cross-react with the KS20 peptide. Both WT and S17E mutations of SG20 peptides were able to stimulate these T cells; however, S17E significantly enhanced T-cell activation as expected (**Figure 5B**). We then used different concentrations of synthetic peptide SG20S17E to stimulate these T-cell hybridomas, and the results showed that 0.1–1 μg/mL SG20S17E could activate all of them (**Figure 5C**). These results confirmed that the CGRP-reactive T cells are present in NOD mice, and IA^g7^-SG20S17E tetramer is an efficient tool to detect CGRP-reactive T cells *in vivo*.

### TRAV and TRBV gene segment usages of CGRP-specific T-cell hybridomas

**Figure 6:**
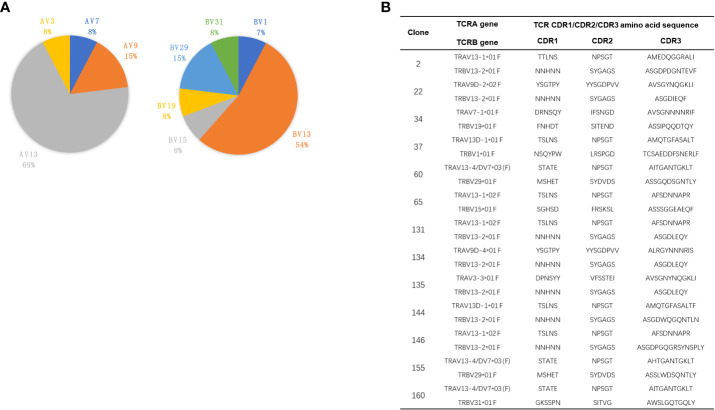
TCR V gene usages of CGRP specific T cell hybridomas. **(A)** Pie charts showed the frequency of TRAV (left) and TRBV (right) gene segment usage of 13 CGRP specific T cell hybridomas. **(B)** The paired CDR1, CDR2, and CDR3 amino acid sequences of TRAV and TRBV of CGRP specific T cell hybridomas.

Sequencing analysis of these CGRP-reactive T-cell hybridomas showed that TRAV13 and TRBV13 were the most utilized TCR V gene segments, being expressed in 69% and 54% of all CGRP-reactive T-cell hybridomas, respectively (**Figure 6A**). The complementarity-determining region (CDR) 1 and CDR2 loops of the TCR β chain contact the MHC alpha-helices, while the hypervariable CDR3 regions interact mainly with the peptide (29). TCRAV and TCRBV gene segment usages of the CGRP-responsive T-cell hybridomas are summarized in **Figure 6B**. In both TCR α and TCR β chains, CDR3 loops have the highest sequence diversity and are the principal determinants of receptor binding specificity. After alignment of all these TRAV13 CDR3 and TRBV13 CDR3 amino acids sequences, we found the common amino acid sequence motifs in CDR3 regions. For example, 131 and 146 T-cell hybridomas share the same TRAV and TRBV usage, while CDR3 sequences of TRBV are different. 60, 155, and 160 TCRs share the same TRAV usage, while TRBV usage is different (**Supplementary Figure S5A**). 131,134, and 135 TCRs share the same CDR3 loops in TCR Vβ regions. Gln in all CDR3 regions of TRBV13 and Asn in the most CDR3 region of TRAV13 are highly conserved, indicating that these amino acids may interact with SG20 peptide similarly (**Supplementary Figure S5B**).

### Human AG20 peptides cross-react with CGRP-reactive T-cell hybridomas

**Figure 7:**
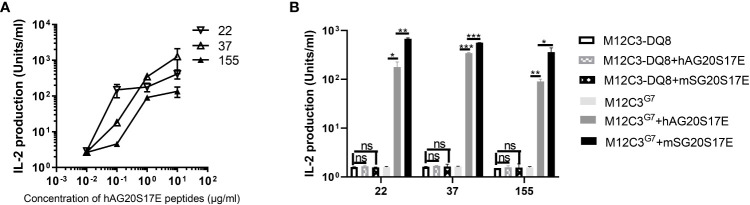
Human AG20 peptide cross-reacted with CGRP reactive T cell hybridomas. **(A)** M12C3^g7^ cells incubated with different concentrations of human AG20S17E peptide stimulated mouse CGRP reactive T cell hybridomas 22, 37, and 155. **(B)** The three representative CGRP reactive T cell hybridomas were stimulated with M12C3-DQ8 incubated with 1 μg/mL hAG20P17E or mSG20P17E and M12C3 cells were used as the negative control. Results are the means ± SEM of triplicate wells. *P *P* < 0.05, **P < 0.01, ***P < 0.001.

The high sequence homology between SG20 and human AG20 suggested that AG20 peptides may cross-react with these CGRP-reactive T cells (**Figure 1**). We stimulated these T-cell hybridomas with M12C3^G7^ loading with different concentrations of human AG20 peptide. The results confirmed that the human CGRP AG20 peptide could be presented by mouse IA^g7^ molecules and cross-react with these CGRP-reactive T-cell hybridomas (**Figure 7A**). HLA-DQ8 molecules not only share structural similarities with the mouse homolog IA^g7^ but can also cross the species barrier and functionally replace IA^g7^ molecules to stimulate diabetogenic T cells and produce diabetes (29). Meanwhile, we tested if HLA-DQ8/AG20 could activate these mouse CGRP-reactive T-cell hybridomas. The M12C3-DQ8 cells incubated with different concentrations of human AG20 and SG20 were used to stimulate CGRP-reactive T-cell hybridomas. HLA-DQ8/AG20 did not activate the mouse CGRP-reactive T-cell hybridomas. This implied that MHC molecules were crucial for CGRP T-cell activation besides the disulfide loops (**Figure 7B**).

## Discussion

The highest risk factor of developing T1D was closely linked to polymorphisms in MHCII genes (36–39). IA^g7^ is the only MHC class II molecule expressed in NOD mice. Like the highest T1D genetic risk factor, HLA-DQ8 and IA^g7^ shared a single polymorphism of a β57 in the β chain (5–7). The β57 polymorphism accounted for the pathogenic peptides that bind to these MHCIIs by breaking a stabilizing salt bridge to α76Arg in the MHCII α chain. The position β57 of IA^g7^ controls the early anti-Ins response and onset of diabetes in NOD mice (40). Ins, ChgA, and IAPP are three well-studied β-cell secretory granule proteins that induced the activation of pathogenic CD4 T cells in NOD mice. NMR structure and molecular simulation revealed that IAPP contains an N-terminal disulfide bond (41–43). The Cryo-EM structure of human CGRP receptors in complex with CGRP also discovered an N-terminal disulfide loop between C2 and C7 (44). Due to the size of the disulfide bond in the KS20 peptide, we anticipated that the similar N-terminal portions of CGRP family proteins could be presented to IAPP-specific T cells in a similar fashion. The mutational study of polymorphic amino acids between SG20 and KS20 peptides showed that A8V, Q10H, and F15L were important for the BDC-5.2.9 TCR recognition. The A8V, Q10H, and F15L amino acids are not part of the N-terminal disulfide loop (**Figure 1**). This suggested that C-terminal amino acids were also critical for BDC-5.2.9 activation in addition to the disulfide bond. Interestingly, KS20V17S mutant can significantly enhance the activation of BDC-5.2.9 compared with WT KS20 (**Figure 3C**). This is in agreement with the fact that KS20 V17 is an important anchoring amino acid in IA^g7^, and Ser was more preferable in the P9 position by forming a hydrogen bond with α76Arg (26, 31). The crystal structure of IA^g7^ showed that the β57Ser forms a hydrogen-bonding network with α76Arg involving an acidic P9 peptide anchor residue (45). Although, IA^g7^-SG20 and IA^g7^-KS20 both have a disulfide loop at N termini, the KS20 peptide cannot cross-react with CGRP-reactive T-cell hybridomas (**Figure 5A, B**). Thus, IAPP and CGRP peptides stimulated non-overlaying CD4 T-cell repertoires, suggesting a novel class of antigen-specific T cells in NOD mice.

A previous study showed that the pathogenic autoreactive CD4 T cells target self-antigen epitopes bound to IA^g7^ in an unfavorable register (25). Both WT SG20 and SG20S17E peptides can activate CGRP-reactive T cells. However, compared with the WT SG20 peptide, SG20S17E mutation significantly strengthened the CGRP-reactive T-cell activation (**Figure 5B**). The results confirmed that poor binding is due to incompatibility between the S17 amino acid of SG20 peptide and IA^g7^ P9 pocket. This is consistent with Ins B:9-23 T-cell activation data, where the mutating of P9 anchoring amino acids to Glu greatly enhances the T-cell responses by forming a hydrogen bond with α76Arg (26, 28). The poor binding may prevent sufficient presentation of SG20 peptides and allow SG20-specific T cells to escape negative selection. The tetramer binding experiments confirmed that the IA^g7^ covalently linked with S17E variation of SG20 peptide tetramer was a critical tool in the detection of SG20 reactive T cells *in vivo* (**Figure 5A**). The CGRP-reactive T cells, including Tregs and effector T cells (Teffs), were present in both prediabetic and diabetic pancreas of NOD mice. These results suggested that high expression of CGRP or modification of CGRP in the pancreas might overcome its poor presentation by IA^g7^, permitting pathogenic CGRP-specific T cells to activate and initiate autoimmunity. Just like KS20-reactive T cells (22), it was noticed that the percentages of CGRP-reactive T cells in the pancreases of diabetic NOD mice were significantly increased as compared with prediabetic NOD mice. In contrast, there were no significant changes in the percentages of 2.5HIP (fusion of a C-peptide fragment to WE14) and Ins-reactive T cells in the pancreas between 10-week-old and diabetic NOD mice (46). Ins and ChgA are produced by β cells of the pancreas, while CGRP is present in the endocrine cells of the human pancreas and nerves (47). In diabetic NOD mice, β cells cannot secret Ins because of the destruction, which leads to no significant increase in Ins-reactive T cells compared with prediabetic NOD mice. ChgA- and Ins-reactive T cells leave the pancreas as the source of antigen decreases *via* the destruction of β cells. However, unlike ChgA- and Ins-reactive T cells, CGRP-specific T cells are still present even after β-cell destruction. The presence of CGRP-specific T cells seemed uniquely correlated with T1D progression and could potentially serve as a useful biomarker. CGRP is also secreted in nerves besides β cells, which may explain the increase in the proportion of CGRP-reactive T cells in the pancreas of diabetic NOD mice compared with prediabetic NOD mice due to inflammation.

Previous findings demonstrated that CGRP may impair Ins secretion and induce Ins resistance in non-Ins-dependent diabetes (48). Here, we displayed that CGRP-reactive CD4 T cells could infiltrate the pancreas before 12 weeks and retain in the pancreas throughout the progression of T1D. For the first time, we generated several CGRP-reactive T cells and determined the TCR V segment usages. Both TCR Vα and Vβ chains are highly skewed towards TRAV13 and TRBV13, respectively. The constrained TCR repertoires could be a consequence of the high immunogenicity of the disulfide bridges of SG20 peptide. Interestingly, some IAPP and CGRP-specific T cells shared the common germline-encoded Vβ segments, such as TRBV15 and TRBV19 (22). This further suggested that the disulfide bridges are extensively interacted by TCRs and could share the same epitopes. The increased amount of CGRP in the pancreas may stimulate the CGRP-reactive T cells, contributing to the destruction of the β cells. Both autoantigen-specific Tregs and Teffs cells are present in the pancreas of NOD mice. CD4^+^ Foxp3^+^ Tregs played an important role in preventing autoimmunity in mice and humans (49). The CGRP-specific Treg cells were detected in the pancreas with the help of tetramers in the prediabetic and diabetic NOD mice, together with Ins P8G-specific and IAPP KS20-specific Tregs in the pancreas. These autoantigen-specific Tregs might play roles in suppressing T1D development (50, 51).

The IAPP peptide formed hybrid peptides with other β-cell antigens, ChgA and C-peptides, and was identified in both mice and humans (10, 52, 53). Like IAPP, other CGRP family proteins could also form hybrid peptides through similar transpeptidation mechanisms (29). HLA-DQ8 shows striking structural similarities with the NOD mouse class II molecule IA^g7^ and has a comparable peptide-binding preference (54, 55). We showed that the human CGRP peptide was able to be presented by IA^g7^ molecules on M12C3 cell surfaces and robustly stimulated mouse CGRP-reactive T cell hybridomas (**Figures 7A, B**). This suggested that the CGRP peptide contains the prominent T-cell epitopes, and CGRP-reactive T cells may also exist in human T1D subjects. Future studies should include the evaluation of the T1D subjects using DQ8-CGRP tetramers. The CGRP-reactive T cells may provide a new predictive biomarker and a new target for future immunotherapy of T1D. Due to the ubiquity of CGRP in the central nervous system and peripheral sensory nerves (13), the CGRP-specific T cells could migrate to the nervous system and be activated by enriched CGRP peptides in the nervous system. These T cells may induce neuroinflammation leading to diabetes-related neuropathic pain, which is the most prevalent long-standing complication of diabetes (56). It would be interesting to see if CGRP-specific T cells are present in the nerve system to confirm that pathogenic CGRP-specific T cells could migrate to other sites and induce inflammation. All CGRP family peptides of mice and humans share a common motif CXXXXC near the N-terminus. Like CGRP and IAPP peptides, the other CGRP family peptides specific T cells could also be associated with the development of T1D in the pancreas and need to be investigated.

## Data availability statement

The original contributions presented in the study are included in the article/**Supplementary Material**. Further inquiries can be directed to the corresponding author.

## Ethics statement

This study was reviewed and approved by National Jewish Health.

## Author contributions

WL and SD designed the studies. WL, RL, YW, YZ, and SD. performed the experiments. WL, RL, MT and SD wrote the paper with help from the other authors. All authors contributed to the article and approved the submitted version.

## Funding

Financial support was provided by National Institutes of Health Grants [T32-AI-074491 (to YW), R56-AI-15348 (to SD), R21-AI-149655 (to SD)], P30-DK-116073 (Colorado Diabetes Research Center), a grant from The ALSAM Foundation (to SD), and the ALSAM Skaggs Scholars Program (to SD).

## Conflict of interest

The authors declare that the research was conducted in the absence of any commercial or financial relationships that could be construed as a potential conflict of interest.

## Publisher’s note

All claims expressed in this article are solely those of the authors and do not necessarily represent those of their affiliated organizations, or those of the publisher, the editors and the reviewers. Any product that may be evaluated in this article, or claim that may be made by its manufacturer, is not guaranteed or endorsed by the publisher.
