# Supplementary material for: Calcitonin gene-related peptide is a potential autoantigen for CD4 T cells in type 1 diabetes
Source: Front Immunol. 2022 Sep 16;13:951281. doi: 10.3389/fimmu.2022.951281 (PMC9523785; doi:10.3389/fimmu.2022.951281)
Supplement: Supplementary file 1 [file Presentation_1.pdf]

Supplementary materials

Calcitonin-gene related peptide is a potential autoantigen for CD4 T cells in type 1 diabetes

Wei Li, Ronghui Li, Yang Wang, Yan Zhang, Munendra S Tomar and Shaodong Dai

Supplementary Table S1. List of primers used in site-directed mutagenesis of KS20 and SG20 peptides

	**Sequence 5' to 3'**	
**Construct name**	**Forward Primer**	**Reverse Primer**
**KS20 K1S**	CTGAGGGCAGCTGCAACACTGCGACGTGTGC	GTGTTGCAGCTGCCCTCAGTCCCGGGGCTGC
**KS20 A8V**	CGACGTGTGTGACTCAACGCTTGGCTAACTT	CGTTGAGTCACACACGTCGCAGTGTTGCATT
**KS20 Q10H**	GTGCTACTCATCGCTTGGCTAACTTTTTGGT	GCCAAGCGATGAGTAGCACACGTCGCAGTGT
**KS20 N14G**	GCTTGGCTGGCTTTTTGGTGAGAAGCTCGGG	ACCAAAAAGCCAGCCAAGCGTTGAGTAGCAC
**KS20 F15L**	TGGCTAACTTGTTGGTGAGAAGCTCGGGCGG	CTCACCAACAAGTTAGCCAAGCGTTGAGTAG
**KS20 V17S**	ACTTTTTGTCGAGAAGCTCGGGCGGGTCACT	GAGCTTCTCGACAAAAAGTTAGCCAAGCGTT
**SG20 S1K**	CTGAGGGCAAATGCAACACTGCGACGTGTGT	GTGTTGCATTTGCCCTCAGTCCCGGGGCTGC
**SG20 V8A**	CGACGTGTGCGACTCATCGCTTGGCTGGCTT	CGATGAGTCGCACACGTCGCAGTGTTGCAGC
**SG20 H10Q**	GTGTTACTCAACGCTTGGCTGGCTTGTTGGA	GCCAAGCGTTGAGTAACACACGTCGCAGTGT
**SG20 G14N**	GCTTGGCTAACTTGTTGAGCAGAAGCGGGG	CTCAACAAGTTAGCCAAGCGATGAGTAACAC
**SG20 L15F**	TGGCTGGCTTTTTGAGCAGAAGCGGGGGCGG	CTGCTCAAAAAGCCAGCCAAGCGATGAGTAA
**SG20 S17V**	GCTTGTTGGTGAGAAGCGGGGGCGGGTCACT	CCGCTTCTCACCAACAAGCCAGCCAAGCGAT

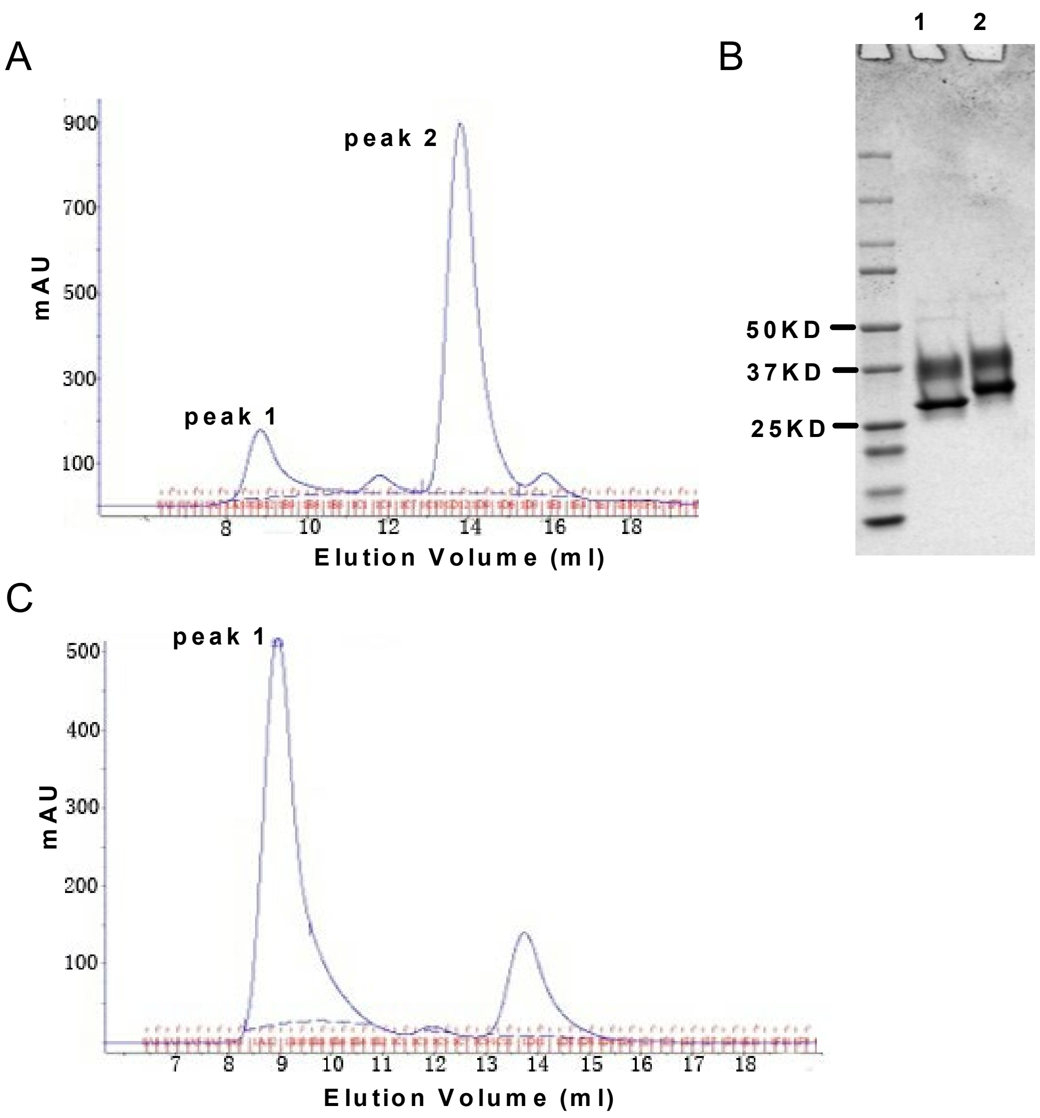

Supplementary Figure 1. Purification of the biotinylated IA^{g7}-SG20 protein and tetramers. **(A)**The biotinylated IA^{g7}-SG20 protein were concentrated to run Superdex 200 Increase gel filtration column. **(B)** The SDS-PAGE was used to evaluate the purity of the biotinylated protein in peak 2: lane 1, non-reduced IA^{g7}-SG20; lane 2, reduced IA^{g7}-SG20. **(C)** Shown are the Superdex-200 Increase chromatography profile of IA^{g7}-SG20 tetramers, peak 1.

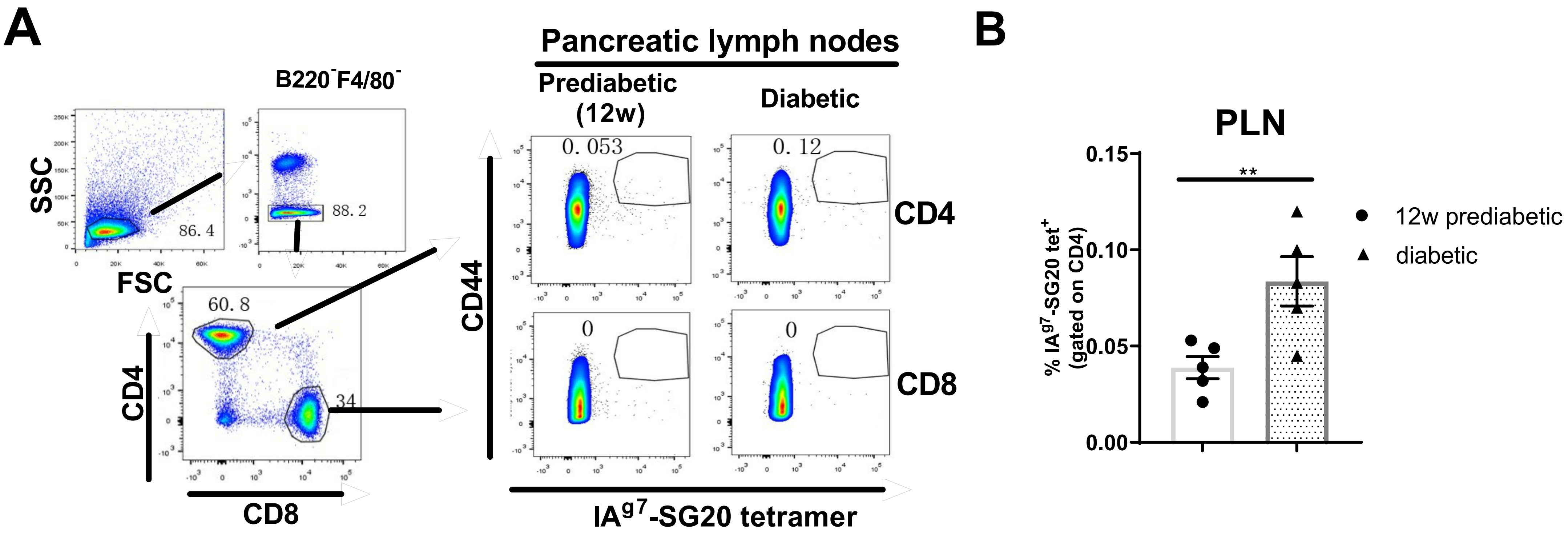

Supplementary Figure 2, related to Figure 4. IA^{g7}-SG20 tet^{+} in the PLNs of prediabetic and diabetic NOD mice. Methods were the same as described in Fig.4. Single-cell suspensions of PLN cells were stained with antibodies and tetramers as the pancreas. **(A)** Representative scatter diagram of IA^{g7}-SG20 tet^{+}CD44^{+} cells in pancreas of different groups. **(B)** The percentages of IA^{g7}-SG20 tet^{+}CD44^{+} in CD4^{+} T cells in prediabetic and diabetic groups. Each symbol represents an individual mouse. $*P < 0.05$, $** P < 0.01$.

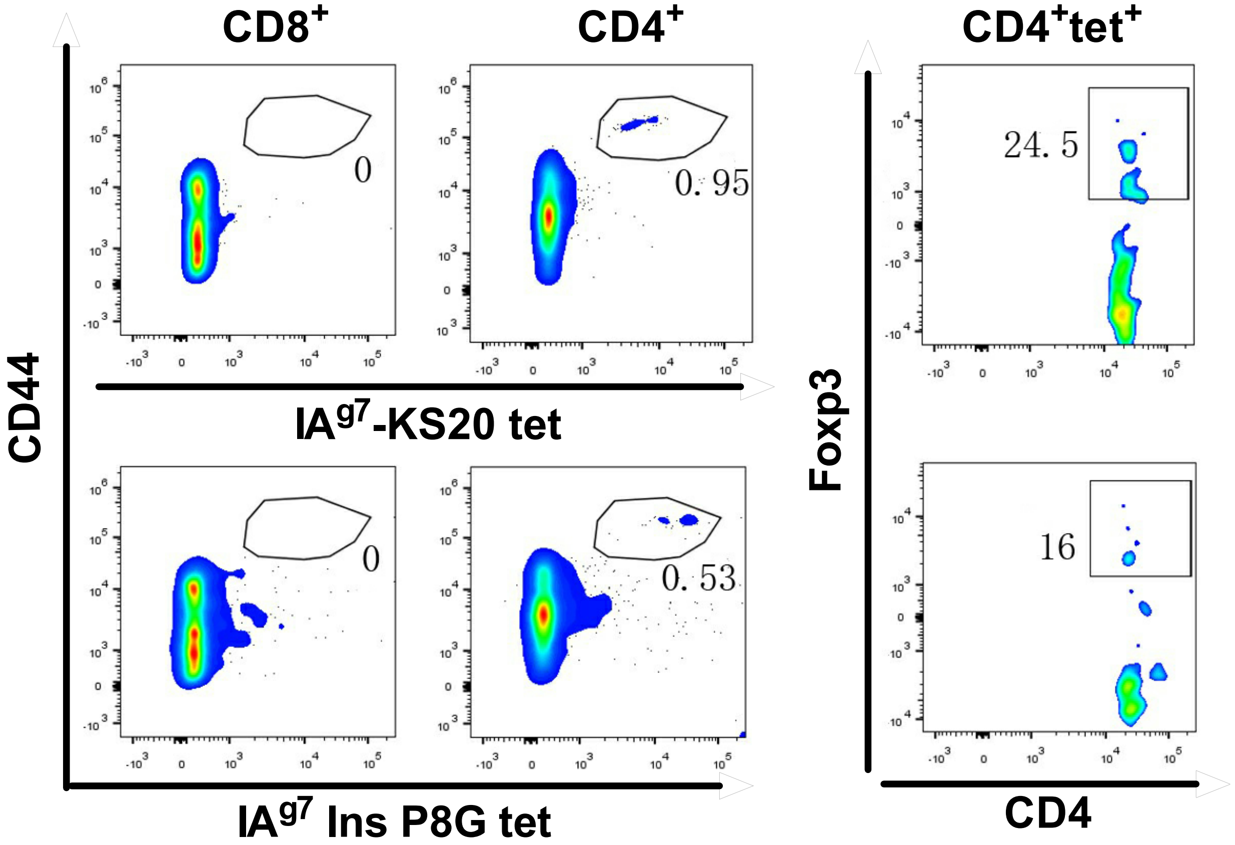

Supplementary Figure 3. KS20 and Insulin P8G reactive T cells were stained with tetramers in diabetic NOD mouse. Pooled pancreatic cells were isolated from diabetic NOD mice and stained with the IA^{g7}-KS20 tetramer and IA^{g7}-ins P8G tetramer separately. Then the cells were stained with anti-B220, anti-F4/80, anti-CD44, anti-CD4, and anti-CD8, anti-Foxp3 and anti-CD25. For tetramer analysis, cells were pregated on B220^{-}, F4/80^{-}, CD8^{-}, CD4^{+}, and CD44^{+} high cells. The tetramer-positive CD4^{+} T cells were divided into two subsets, Foxp3^{+} and Foxp3^{-} T cells.

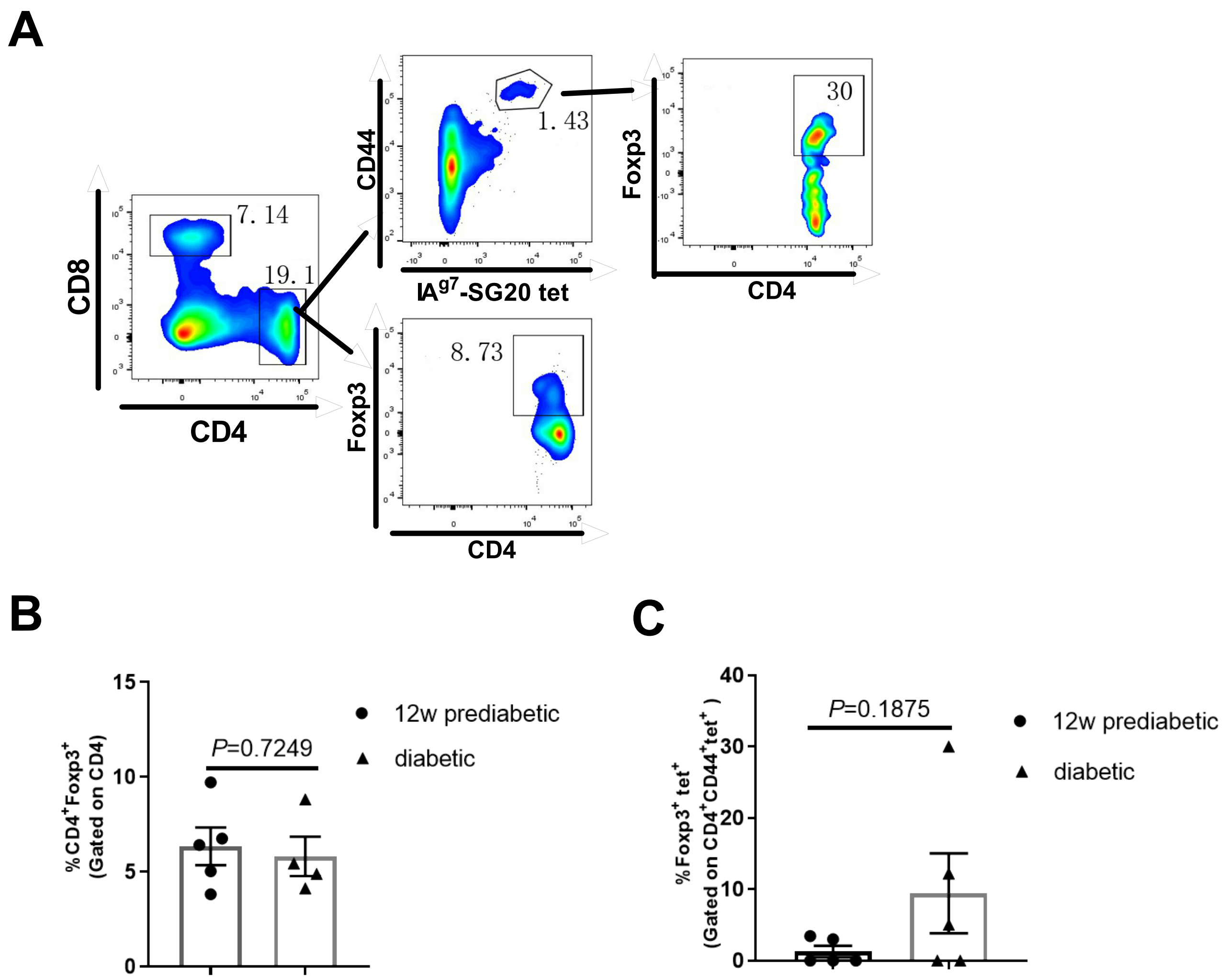

Supplementary Figure 4, related to Figure 4. CGRP reactive Treg cells are present in the pancreas of prediabetic and diabetic NOD mice. Single-cell suspensions were prepared from the whole pancreas of each prediabetic (12w) or diabetic NOD mice, stained with IA^{g7}-SG20 tetramers, anti-B220, anti-F4/80, anti-CD44, anti-CD4, anti-CD8, and anti-Foxp3. For CGRP-specific Tregs analysis, cells were pregated on live, $B220^{-}$, $F4/80^{-}$, $CD8^{-}$, $CD4^{+}$, and $CD44^{high}$ cells, and then analyzed by flow cytometry. **(A)** Representative scatter diagram of IA^{g7}-SG20 $tet^{+}CD44^{+}$ $Foxp3^{+}$cells in different groups. **(B and C)** The percentages of $CD4^{+}Foxp3^{+}$ Tregs **(B)** and IA^{g7}-SG20 $tet^{+}CD44^{+}$ Tregs **(C)** in the pancreas were analyzed. Each symbol represents an individual mouse.

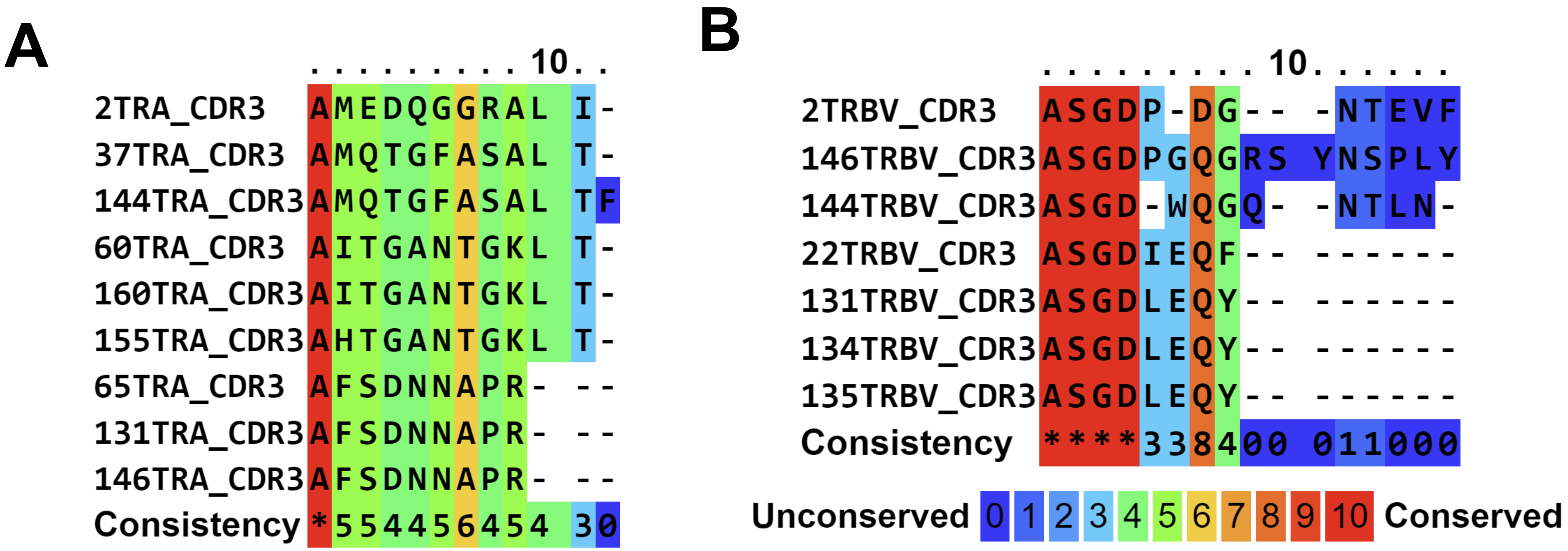

Supplementary Figure 5. The CGRP specific TCR CDR3 amino acid sequence alignment of TRAV13 and TRBV13 using PRALINE.
